# Clinical Risk Factors for Central Line-Associated Venous Thrombosis in Children

**DOI:** 10.3389/fped.2015.00035

**Published:** 2015-05-05

**Authors:** Samir H. Shah, Alina Nico West, Robert J. Sepanski, Debbie Hannah, William N. May, Kanwaljeet J. S. Anand

**Affiliations:** ^1^Department of Pediatrics, University of Tennessee Health Science Center, Le Bonheur Children’s Hospital Memphis, Memphis, TN, USA; ^2^Children’s Foundation Research Institute, Le Bonheur Children’s Hospital, Memphis, TN, USA; ^3^Department of Performance Improvement and Patient Safety, Children’s Hospital of The King’s Daughters, Norfolk, VA, USA; ^4^Department of Quality Improvement, Le Bonheur Children’s Hospital, Memphis, TN, USA; ^5^Le Bonheur Children’s Hospital, Memphis, TN, USA

**Keywords:** pediatric critical illness, central venous line thrombosis, pediatric thrombosis risk factors, clinical outcomes of pediatric thrombosis, pediatric thrombosis morbidity

## Abstract

**Background:**

Identifying risk factors related to central venous line (CVL) placement could potentially minimize central line-associated venous thrombosis (CLAVT). We sought to identify the clinical factors associated with CLAVT in children.

**Methods:**

Over a 3-year period, 3733 CVLs were placed at a tertiary-care children’s hospital. Data were extracted from the electronic medical records of patients with clinical signs and symptoms of venous thromboembolism, diagnosed using Doppler ultrasonography and/or echocardiography. Statistical analyses examined differences in CLAVT occurrence between groups based on patient and CVL characteristics (type, brand, placement site, and hospital unit).

**Results:**

Femoral CVL placement was associated with greater risk for developing CLAVT (OR 11.1, 95% CI 3.9–31.6, *p* < 0.0001). CVLs placed in the NICU were also associated with increased CLAVT occurrence (OR 5.3, 95% CI 2.1–13.2, *p* = 0.0003). CVL brand was also significantly associated with risk of CLAVT events.

**Conclusion:**

Retrospective analyses identified femoral CVL placement and catheter type as independent risk factors for CLAVT, suggesting increased risks due to mechanical reasons. Placement of CVLs in the NICU also led to an increased risk of CLAVT, suggesting that small infants are at increased risk of thrombotic events. Alternative strategies for CVL placement, thromboprophylaxis, and earlier diagnosis may be important for reducing CLAVT events.

## Introduction

The incidence of venous thromboembolism (VTE) is increasing in the hospitalized pediatric population (1–4). This is surprising due to the previously described rarity of this potentially fatal condition (5). Although variable across institutions, an overall VTE incidence of 188/100,000 hospitalized pediatric patients was found in the retrospective KID database (3, 6). The overall incidence of VTE may have increased recently, since there are no current established guidelines for pediatric thromboprophylaxis (4, 7, 8).

A multifactorial process leads to VTE development in hospitalized children, possibly related to their age and race, primary diagnoses, congenital and genetic factors, and mechanical factors (3, 4, 9). Retrospective and prospective studies have identified higher risks for VTE in children below 1 year of age, adolescents, children with specific renal, hematologic, infectious, traumatic, and oncologic diagnoses, and those exposed to mechanical factors (1, 4, 7, 10, 11). Beck et al. found sonographic evidence of venous thrombosis in 18.3% of children requiring a central venous line (CVL) (11).

Central venous line insertion is associated with multiple complications including central line-associated bloodstream infections (CLABSI) and thrombosis (12, 13). Although CLABSI is well characterized in pediatric and adult hospitalized patients, VTE development is less studied in the pediatric population (1, 14). To date, there are no published guidelines for the diagnosis of central line-associated venous thrombosis (CLAVT) in children (14). The diagnosis of CLAVT has depended upon clinical signs and symptoms, the use of Wells’ criteria, along with imaging studies such as Doppler ultrasound and traditional venography (1, 4).

We conducted a retrospective cohort study to identify risk factors associated with CLAVT incidence in a tertiary-care children’s hospital. We hypothesized that CLAVT identified via ultrasound imaging would be increased in children <1 year of age and those with femoral CVL placement.

## Materials and Methods

### Data collection

With Institutional Review Board approval, data were collected on 3733 CVLs placed within a 3-year period (2010–2012). The number of charts reviewed did include placement of multiple central lines in the same patient. “Symptomatic” CLAVT was defined as thrombosis associated with a blocked or leaking CVL, progressive distal limb edema, or clinical signs of pulmonary embolism (chest pain, shortness of breath, tachycardia, tachypnea, hypoxia, hemoptysis). Standard nursing assessment of either the presence or absence of venous thrombosis was done every 12 h and CLAVT was characterized by redness, edema, and presence/absence of a distal pulse. CLAVT was confirmed via either Doppler ultrasonography (with increased echogenicity at the site or presence of a filling defect) or with presence of a thrombus on echocardiography. Patients with venous thrombosis either present at hospital admission or unrelated to CVL placement were excluded. The following variables were included hospital location of catheter placement, catheter type, CVL placement site, number of attempts to place CVL, and use of ultrasonography for vascular access.

### Statistical analyses

In order to adjust for bias associated with low occurrence of CLAVT in many of the subcategories being examined, the Firth method of multiple logistic regression based on a penalized maximum likelihood function was used to analyze associations between CLAVT and line type (Arrow™, Cook™, Broviac™, PICC, and All Other), CLAVT and unit of insertion (OR, PICU, NICU, All Other), and CLAVT and anatomical insertion site (Femoral, Jugular, Subclavian, Upper Extremity, and All Other). “All other” subcategories represented groupings of all but the categories that were most common in each dataset.

## Results

The numbers of CLAVT events are listed in Table 1. The association of total number of central lines placed with the number of CLAVT events shows a slight decline of events in 2011 and 2012, in comparison to data shown for 2010. The number of CLAVT events per total annual catheter number increased in the PICU compared to other hospital locations at the end of the 3-year period (Table 2). Although in 2010, the NICU had the highest percentage of CLAVT events. Additionally, CVL placement in the NICU had a higher odds ratio than other units (OR 5.3, 95% CI 2.1–13.2, *p* = 0.0003; Table 3).

**Table 1:** **Number of CLAVT Events in a single tertiary pediatric hospital**.

	2010	2011	2012
Total number of central lines	946	1439	1348
CLAVT	20	21	21
Patients with 2 CLAVT events	2	0	N/A^a^

*^a^Data from 2012 was de-identified*.

**Table 2:** **Percentage of CLAVT events per hospital unit^a^**.

Year	PICU	CVICU	NICU	Hospital-wide
2010	2.98	0	7.41	2.11
2011	3.53	3.85	1.40	1.46
2012	4.03	2.04	0.79	1.56

*^a^PICU, pediatric intensive care unit; CVICU, cardiovascular intensive care unit; NICU, neonatal intensive care unit*.

**Table 3:** **Multiple logistic regression analysis of 3733 central venous lines^a^**.

Multiple logistic regression results^a^ (dependent variable = CLAVT occurrence)				
**Independent variable**	**Subcategory**	**CLAVT lines/all lines in subcategory**	**Adjusted odds ratio (95% CI)**	***p***

CVL line type	PICC (reference)	5/1227	1.0 (–)	–
	Arrow™	11/541	4.2 (0.9–18.8)	0.06
	**Broviac™**	**6/325**	**8.9 (1.9–41.9)**	**0.006**
	**Cook™**	**33/742**	**4.2 (1.0–17.2)**	**0.05**
	All other line types	7/898	3.8 (0.8–17.6)	0.09
Unit (CVL placement)	OR (reference)	13/1279	1.0 (–)	–
	**NICU**	**11/377**	**5.3 (2.1–13.2)**	**0.0003**
	PICU	27/757	1.7 (0.8–3.8)	0.17
	All other units	11/1320	1.0 (0.4–2.2)	0.95
CVL insertion site	Subclavian (reference)	5/931	1.0 (–)	–
	**Femoral**	**37/473**	**11.1 (3.9–31.6)**	**<0.0001**
	Jugular	7/502	2.4 (0.7–7.7)	0.15
	Upper extremity	3/832	1.7 (0.3–11.2)	0.58
	All other sites	10/995	1.9 (0.7–5.5)	0.23

*^a^Using Firth’s method of penalized maximum likelihood*.

*Reference subcategories were chosen as ones having the lowest frequency of CLAVT occurrence from among those having the highest frequency of CVL’s. Subcategories showing an association with CLAVT that differs significantly from the reference subcategory are highlighted in bold*.

Risk factor assessment (Table 3) showed that femoral vein placement contributed significantly to CLAVT (OR 11.1, 95% CI 3.9–31.6, *p* < 0.0001), in comparison to upper extremity lines. Broviac™ and Cook™ catheters were also more commonly associated with CLAVT (Table 3).

## Discussion

Central line-associated venous thrombosis is a significant iatrogenic complication of critical illness in our tertiary-care facility. Significant risk factors at our facility include anatomical site, hospital location of CVL placement, and types of lines placed. Variability exists in the diagnosis of CLAVT and its incidence based on clinical risk factors (6). Our finding that small children, specifically NICU patients, have the highest number of CLAVT events is consistent with previous studies of children with central venous catheters (1, 3, 4, 7, 8, 11).

Central venous line characteristics, including type, brand, and anatomic site of insertion and their relationship to number of CLAVT events were evaluated in this study. Our data support the well-established postulate that femoral CVLs predispose pediatric patients to CLAVT, especially in hospitalized children below 1 year of age (4, 15). Higgerson et al. found that femoral DVTs occur in mechanically ventilated PICU patients, whether or not they were CVL-related (7). We found that the highest number of CLAVTs occurred in PICU patients, with a slight decrease in the number of line events in 2012, likely due to a Hawthorne effect. Nonetheless, our findings are congruent with these previously published data, both suggesting that CLAVT is a growing problem in the PICU population. Other CVL characteristics – CVL type and brand – did not have a significant impact on CLAVT development in a study by Male et al. (16). Broviac™ and Cook™ catheters were significantly associated with CLAVT risk in our study; however, a larger, prospective study is needed to assess this risk factor more precisely.

Overall, the incidence of CLAVT minimally increased over a 3-year period. Increased awareness of the study itself could have lead to increased diagnosis in 2011 and 2012. Also, in our patients with symptomatic CLAVT, the probability of screening could potentially be higher (17).

There are several limitations of this retrospective study. CLAVT is associated with increased mortality and increased length of stay, especially in the PICU, but we did not have adequate sample size to examine these outcomes in our study (1, 4, 7, 18). Considering the association of CLAVT with increased mortality, it would have been prudent to assess levels of critical illness. Only information on symptomatic VTEs, based on clinical signs and symptoms, was captured in this study and confirmed by the use of Doppler ultrasonography or echocardiography. Geerts et al. assert that the ratio of asymptomatic to symptomatic DVT ranges from 5:1 to 10:1 and that asymptomatic DVT evolves to symptomatic VTE (17). Asymptomatic CLAVTs were probably undetected in our patients and therefore, we will evaluate CLAVT prospectively in order to gather more data including diagnostic methods and primary diagnoses. CLABSI association was not addressed in this study, as our dataset was limited. Lastly, the use of thromboprophylaxis was not evaluated during this study. Guidelines for use of thromboprophylaxis in pediatric patients have been based upon adult guidelines (19). It is essential to evaluate thromboprophylaxis prospectively, in order to establish which anti-thrombolytic therapies are most effective in pediatric patients (4).

Despite the limitations of this study, we found that ICU patients with femoral CVL placement had the highest incidence of CLAVT in our facility. Based on increased awareness, it would be possible to reach an earlier diagnosis and evaluation interventions to decrease CLAVT rates. Prospective, multicenter studies are warranted to enhance earlier diagnostic strategies and to determine the most effective thromboprophylaxis and antithrombotic interventions required for PICU patients.

## Conflict of Interest Statement

The authors declare that the research was conducted in the absence of any commercial or financial relationships that could be construed as a potential conflict of interest.
